# Overexpression of δ-catenin is associated with a malignant phenotype and poor prognosis in colorectal cancer

**DOI:** 10.3892/mmr.2015.3918

**Published:** 2015-06-11

**Authors:** HONG ZHANG, SHUN-DONG DAI, SHU-LI LIU, FANG-YUAN ZHANG, CHAO-LIU DAI

**Affiliations:** 1 Department of Colorectal Surgery, Shengjing Hospital, China Medical University, Shenyang, Liaoning 110004, P.R. China; 2 Department of Pathology, The First Affiliated Hospital and College of Basic Medical Sciences, China Medical University, Shenyang, Liaoning 110001, P.R. China

**Keywords:** δ-catenin, colorectal cancer, prognosis

## Abstract

Little is known regarding the expression or clinical significance of δ-catenin, a member of the catenin family, in colorectal cancer (CRC). The present study examined the expression of δ-catenin using immunohistochemistry in 110 cases of CRC, including 70 cases with complete follow-up records and 40 cases with paired lymph node metastases. In addition, δ-catenin mRNA and protein expression were compared in 30 pairs of matched CRC and normal colorectal tissues by reverse transcription quantitative polymerase chain reaction and western blot analysis. δ-Catenin was weakly expressed or absent in the cytoplasm of normal intestinal epithelial cells, whereas positive δ-catenin expression local-ized to the cytoplasm was observed in CRC cells. The rate of positive δ-catenin expression in CRC (68.18%; 75/110) was significantly higher than that in normal colorectal tissues (36.7%; 11/30; P<0.001). In addition, δ-catenin mRNA and protein expression were significantly increased in CRC tissues compared to those in their matched normal tissues (all P<0.05). The expression of δ-catenin in stage III–IV CRC was higher than that in stage I–II CRC, and the expression of δ-catenin in the tumors of patients with lymph node metastases was higher than that in patients without lymph node metastases. Kaplan-Meier survival curves demonstrated that the survival time of patients with positive δ-catenin expression was shorter than that of patients with negative δ-catenin expression (P=0.005). Furthermore, Cox multivariate analysis indicated that the tumor, nodes and metastasis stage (P=0.02) and positive δ-catenin expression (P=0.033) were independent prognostic factors in CRC. The present study therefore indicated that δ-catenin may be a suitable independent prognostic factor for CRC.

## Introduction

Colorectal cancer (CRC) is one of the most common malignant tumor types, and the incidence of CRC is increasing by 2% per year worldwide. Although the prognosis for CRC has greatly improved with the continuous development of diagnostic techniques, the mortality rate remains high (1). The major causes of CRC-associated mortality are recurrence and metastasis. Loss of intercellular adhesions has a vital role during the process of tumor invasion and metastasis. E-cadherin is the major molecule responsible for maintaining intercellular adherens junctions, and loss of E-cadherin expression is closely associated with de-differentiation and metastasis in a variety of tumor types (2,3). E-cadherin can maintain the stability of intercellular adherens junctions by binding with multiple catenins to form the cadherin catenin complex.

δ-Catenin is one member of the catenin family and consists of 10 Armadillo (Arm) repeats. Initially, δ-catenin was considered to only be expressed in cerebral neurons, where it has been shown to bind with presenilin to exert a function in the development of Alzheimer's disease (4–6). However, previous studies have reported a close correlation between the expression of δ-catenin and cancer. In a comparative microarray study of benign prostatic hyperplasia and prostate cancer, Burger *et al* (7) verified that δ-catenin was markedly upregulated at the transcriptional level in prostate adenocarcinoma. Subsequently, Lu *et al* (8) demonstrated that δ-catenin was significantly overexpressed and associated with the Gleason score in prostate cancer. Furthermore, Zhang *et al* (9) reported that δ-catenin is overexpressed in lung cancer tissues and can promote a malignant phenotype in non-small cell lung cancer cells via enhancing the activity of the transcription factor Kaiso (10). These studies indicated that δ-catenin has a role in the initiation and progression of cancer. However, it has remained elusive whether δ-catenin is overexpressed in CRC, or whether the expression of δ-catenin is correlated with the clinicopathological features of CRC.

In the present study, δ-catenin protein expression was determined in 110 cases of CRC using immunohistochemistry, and the correlation between δ-catenin expression and the clinico-pathological features of CRC was investigated. In addition, the expression of δ-catenin in primary tumor foci and lymph node metastases was compared in 40 matched tissues from CRC patients with lymph node metastases. The prognostic value of δ-catenin in the 70 cases of CRC for which complete follow-up data were available was determined. Finally, δ-catenin protein and mRNA expression were compared in 30 paired CRC and adjacent normal tissues, and the correlation between the expression of δ-catenin mRNA and the clinicopathological features of CRC was investigated.

## Materials and methods

### Tissue samples

Formalin-fixed, paraffin-embedded (FFPE) blocks from 110 cases of CRC and 30 normal colorectal tissue specimens were obtained from the Department of Pathology, Shengjing Hospital of China Medical University (Shenyang, China). None of the patients had received radiotherapy, chemotherapy or immunotherapy prior to tumor excision. In total, 67 of the patients were male and 43 were female (1.56:1 male-to-female ratio). The patients' age at the time of surgery ranged from 31 to 87, with an average age of 61 years.

Complete follow-up data were available for 70 of the cases of primary CRC, which were surgically excised between May 2004 and July 2005. In addition, lymph node metastases were present in 40 of the 110 cases. To evaluate the tumor, nodes and metastasis (TNM) stage, at least 12 lymph nodes were obtained during surgical resection. All tumors were classified according to the TNM staging system, as revised by the International Union Against Cancer (UICC) in 2002 (11). All specimens were re-evaluated for diagnosis according to the World Health Organization (WHO) criteria (12) for the classification of colorectal cancer. All 110 of the CRC specimens were adenocarcinomas, with 14 stage-I cases, 39 stage-II cases, 42 stage-III cases and 15 stage-IV cases; furthermore, 36 cases were highly differentiated, 55 cases were moderately differentiated and 19 cases were poorly differentiated.

In addition, paired tumor and non-tumor tissues (>5 cm from the primary tumor edge) were obtained from 30 cases of CRC, immediately frozen in liquid nitrogen and stored at −70°C for RNA and protein analysis. The present study was conducted in accordance with the regulations of and was approved by the Institutional Review Board of China Medical University. Informed consent was obtained prior to surgery from all enrolled patients.

### Immunohistochemical staining and evaluation

FFPE tissue blocks were cut into 4-*µ*m sections and mounted on poly-L-lysine-coated slides (SLI-2002; MaiXin, Fuzhou, China). The sections were de-paraffinized in xylene, re-hydrated using a graded ethanol series, incubated with 3% hydrogen peroxide in methanol for 20 min, and then washed in phosphate buffered saline (PBS) for 5 min (All from MaiXin). Antigen retrieval was conducted using enzymatic antigen retrieval solution for 15 min at room temperature, and then the sections were washed in PBS for 5 min and blocked using normal blocking serum from the Ultrasensitive™ S-P kit (KIT-9720; MaiXin) for 30 min. The sections were incubated with anti-δ-catenin primary antibody (1:400; Abcam, Cambridge, UK) at 4°C overnight. Following washing with PBS, the sections were stained using diaminobenzidine tetrahydrochloride (MaiXin) as a chromogen, lightly counter-stained with hematoxylin (MaiXin), dehydrated and mounted. For the negative controls, the primary antibody was replaced with PBS.

The scoring criteria for δ-catenin were identical to those described by Lu *et al* (8). All sections were assessed by three observers blinded to the study. Cases with discrepancies were jointly re-evaluated by the investigators, and a consensus was obtained. The sections were evaluated at low magnification (×100) with the Olympus IX51 microscope (Olympus America, Inc., Melville, NY, USA) to identify areas in which δ-catenin was evenly stained. A total of 400 tumor cells were counted and the percentage of positively-stained cells was calculated. The proportion of cells exhibiting δ-catenin expression was categorized as follows: 0, <1%; 1, 1–25%; 3, 51–75%; and 4, >75%. The relative staining intensity was categorized as follows: 0 (no staining); 1 (weak); 2 (intermediate) and 3 (strong). The proportion and intensity scores were multiplied to obtain a total score; scores <2 were considered negative, while scores ≥2 were considered positive.

### Reverse transcription quantitative polymerase chain reaction (RT-qPCR)

RT-qPCR was performed following the MIQE guidelines (Minimum Information for Publication of Quantitative Real-Time PCR Experiments) published by Bustin *et al* (13). Total RNA was isolated from the tissues using TRIzol (Invitrogen Life Technologies, Carlsbad, CA, USA) according to the manufacturer's instructions. 1% agarose gel electrophoresis was performed for 30 min to detect the degradation of RNA samples. The concentration and purity of extracted RNA were determined using the GenQuant RNA/DNA calculator (GeneQuant 1300/100; Amersham-Pharmacia Biotech, Cambridge, UK). Deoxyribonuclease (DNase) treatment was performed using DNase I (Invitrogen Life Technologies). 1 *µ*g RNA was reverse transcribed using the high-capacity cDNA RT kit (Applied Biosystems, Thermo Fisher Scientific, Waltham, MA, USA) following the manufacturer's instructions.

Real-time PCR was performed using the ABI Prism 7900HT Fast System (Applied Biosystems) with SYBR Premix Ex Taq II (Takara, Dalian, China). Amplifications were performed in a total volume of 10 *µ*l using the primer sequences from Takara listed in Table I, with an initial dena-turation at 95°C for 30 sec followed by 40 cycles of 95°C for 5 sec and 60°C for 30 sec. GAPDH was used as an internal control. The presence of single PCR amplicons in each reaction was confirmed using melting curve analysis. The data were analyzed using the 2^−ΔCq^ method with the SDS 2.4 software package (Applied Biosystems) following the method of Schmittgen and Livak (14).

### Western blot analysis

Total protein was extracted from the colorectal tissues using lysis buffer (150 mM NaCl, 1% NP-40, 0.1% SDS, 2 mg/ml aprotinin and 1 mM phenylmethanesulfonylfluoride; Beyotime Insititute of Biotechnology, Shanghai, China) for 30 min at 4°C, the lysates were centrifuged at 14,000 ×g for 30 min at 4°C and the supernatants were collected. Aliquots containing 50 mg protein were separated by 8% SDS-PAGE (Beyotime Institute of Biotechnology) and transferred to PVDF membranes (Merck Millipore, Darmstadt, Germany) at 100 V for 2.5 h at 4°C. The protein concentration was determined using the bicinchoninic acid assay (Beyotime Institute of Biotechnology). The membranes were blocked in 5% skimmed milk for 2 h, and the proteins were detected by incubation with mouse monoclonal antibodies against δ-catenin (ab54578; 1:500; Abcam, Cambridge, UK) or GAPDH (E021010-01; 1:1,000; EarthOx, San Francisico, CA, USA) overnight at 4°C, followed by anti-mouse immunoglobulin G conjugated to horse radish peroxidase at a dilution of 1:2,000 for 2 h at room temperature. The bands were visualized using the EC3 Imaging System (UVP LLC, Upland, CA, USA) and the optical densities were normalized to GAPDH using ImageJ software, version 1.44 (NIH, Bethesda, MD, USA).

### Statistical analysis

All *in vitro* experiments were performed at least three times and values are expressed as the mean ± standard deviation. Pearson's χ^2^ test was used to analyze the correlation between δ-catenin expression and clinicopatho-logical features of CRC. The overall survival probabilities were calculated using the Kaplan-Meier method and compared using the log-rank test. To determine the significant factors associated with overall survival, a multivariate Cox proportional hazard model was created. All statistical analyses were performed using SPSS 13.0 for Windows (SPSS Inc., Chicago, IL, USA). P<0.05 was considered to indicate a statistically significant difference between values.

## Results

### δ-Catenin is overexpressed and associated with poor prognosis in CRC

**Figure 1:**
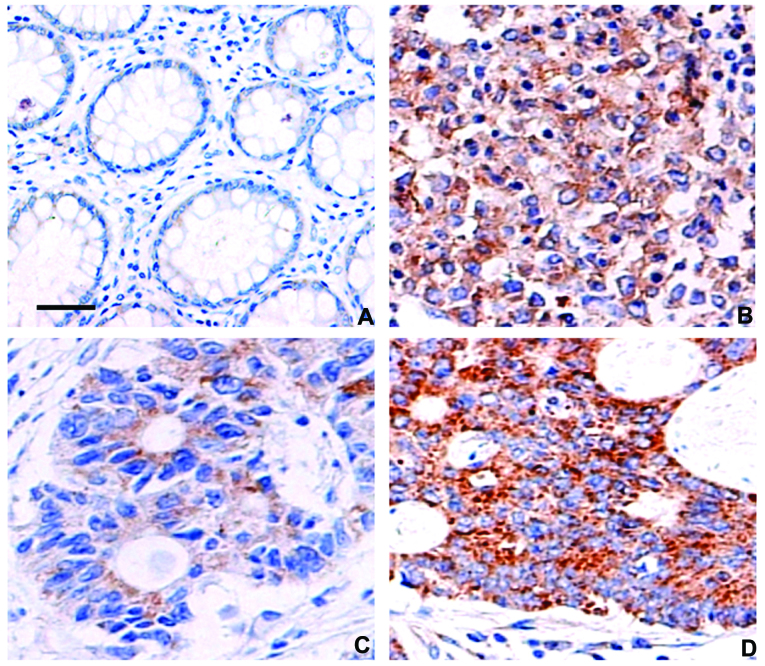
Immunohistochemical staining for δ-catenin in normal colorectal tissues and colorectal cancer tissues. (A) Weak expression of δ-catenin was observed in the cytoplasm of normal colorectal gland epithelial cells. Scale bar, 50 *µ*m. (B and C) Obviously enhanced expression of δ-catenin was observed in (B) the cytoplasm of poorly differentiated colorectal cancer cells and (C) highly differentiated colorectal cancer cells. (D) The expression of δ-catenin was also higher in lymph node metastases, compared to the corresponding primary tumor foci in C. Magnification, ×400.

Negative or weak δ-catenin immunoreactivity was observed in the cytoplasm of normal colorectal mucosal epithelial cells (Fig. 1A). Based on the scoring criteria, all of the normal colorectal mucosa sections were scored as negative. Varying degrees of δ-catenin-positive staining were observed in CRC, mainly in the cytoplasm of the tumor cells (Fig. 1B and C). The rate of positive δ-catenin expression in CRC (75/110; 68.18%) was significantly higher than that in normal colorectal tissues (11/30, 36.7%; P<0.001).

There was no significant association between positive δ-catenin expression and the age, gender, lesion location or tumor size in CRC patients (P>0.05); however, positive δ-catenin expression was closely associated with the degree of differentiation, TNM stage and lymph node metastasis in CRC (Table II). The rates of positive δ-catenin expression in poorly differentiated CRC (18/19; 94.74%), moderately differentiated CRC (36/55; 65.45%) and well-differentiated CRC (21/36; 58.33%) were significantly different (P=0.012 according to Fisher's probabilities test). The rate of positive δ-catenin expression in stage-I–II CRC (56.6%; 30/53) was significantly lower than that in stage-III–IV CRC (78.95%; 45/57; P<0.05). The rate of positive δ-catenin expression in the tumors of patients with lymph node metastasis (79.25%; 42/53) was significantly higher than that in patients without lymph node metastasis (57.89%; 33/57; P= 0.016). In the 40 cases of CRC with matched lymph node metastases specimens, the expression rate of δ-catenin in lymph node metastases (92.5%; 37/40; Fig. 1D) was significantly higher than that in the corresponding primary tumor foci (75%; 30/40; P=0.002; Fig. 1C).

**Figure 2:**
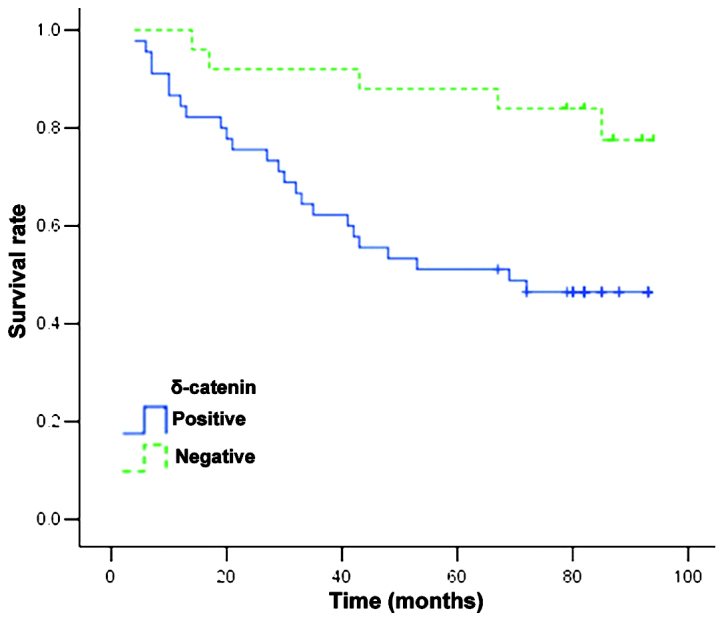
δ-Catenin has a prognostic value in colorectal cancer. Kaplan-Meier survival curves for colorectal cancer patients stratified by the expression of δ-catenin. The mean survival time and five-year survival rate for colorectal cancer patients with positive δ-catenin expression (blue solid line; n=45) were significantly lower than those of patients with negative δ-catenin expression (green dashed line; n=25; P=0.005).

For the 70 CRC patients with complete follow-up data, the five-year survival rate was 64.29% (45/70). Kaplan-Meier survival analysis demonstrated that the mean survival time of patients with positive δ-catenin expression was significantly shorter than that of patients with negative δ-catenin expression (84.02±4.63 vs. 58.53±5.22 months; Log-Rank test P=0.005; Fig. 2), indicating that positive expression of δ-catenin may be indicative of a poor prognosis in CRC patients. The Cox model multivariate analysis confirmed that the TNM stage (P= 0.009) and positive expression of δ-catenin (P=0.031) were independent risk factors, which affected the prognosis of patients with CRC (Table III).

### Expression of δ-catenin mRNA and protein are significantly upregulated in CRC

**Figure 3:**
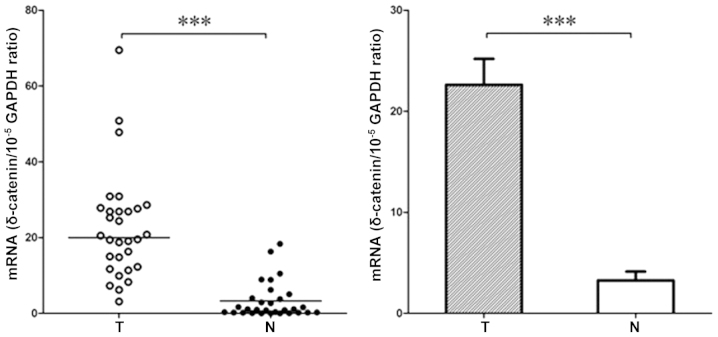
Reverse transcription quantitative polymerase chain reaction analysis of δ-catenin mRNA expression in colorectal cancer tissues. δ-Catenin mRNA expression was significantly higher in colorectal cancer tissues than the matched normal colorectal tissues. The horizontal solid lines indicate the median value in each group. In the bar graph, values are expressed as the mean ± standard deviation. ^***^P<0.0001 (n=30). T, tumor tissue; N, normal tissue.

The expression of δ-catenin mRNA was assessed by RT-qPCR in 30 paired colorectal cancer specimens and the adjacent normal colorectal tissues. The relative δ-catenin mRNA expression levels were seven-fold higher in CRC tissues (22.61±2.57) than those in the matched normal colorectal tissues (3.25±0.88; t=7.137, P<0.0001; Fig. 3).

**Figure 4:**
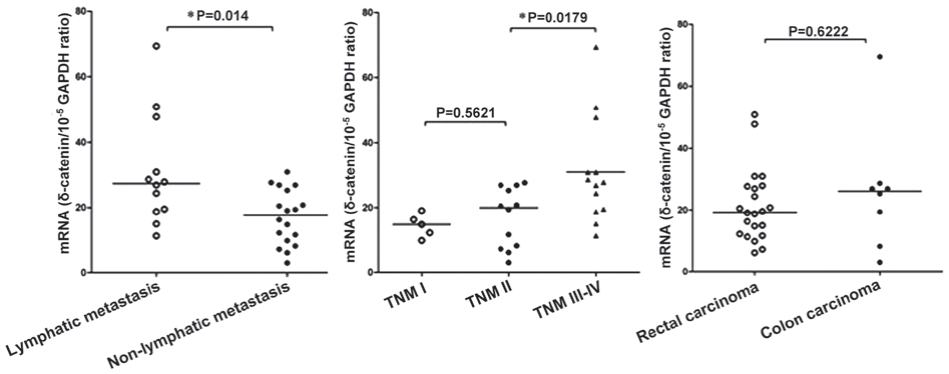
Correlation between δ-catenin mRNA expression and the clinicopathological features of 30 colorectal cancer patients. The horizontal solid lines indicate the median value in each group. ^*^P-values were calculated using the non-parametric Mann-Whitney test or Kruskal-Wallis test. TNM, tumor, nodes, metastasis stage.

The correlation between δ-catenin mRNA expression and the clinicopathological features of CRC was examined in 30 patients (Fig. 4). A significant correlation was observed between the expression of δ-catenin mRNA and lymph node metastasis, as the δ-catenin mRNA expression levels were 1.8-fold higher in patients with lymph node metastasis (30.93±4.89) than those in patients without metastasis (17.06±1.971; P=0.014). Furthermore, a positive correlation was observed between TNM stage and δ-catenin mRNA expression. The Kruskal-Wallis test revealed that the δ-catenin mRNA expression levels were significantly different in CRC tissues at different TNM stages (P=0.0116), with the most significant difference observed between stage III–IV and stage I–II (P=0.0179). There was no correlation between the δ-catenin mRNA expression levels and tumor location, consistent with the immunohistochemistry results.

**Figure 5:**
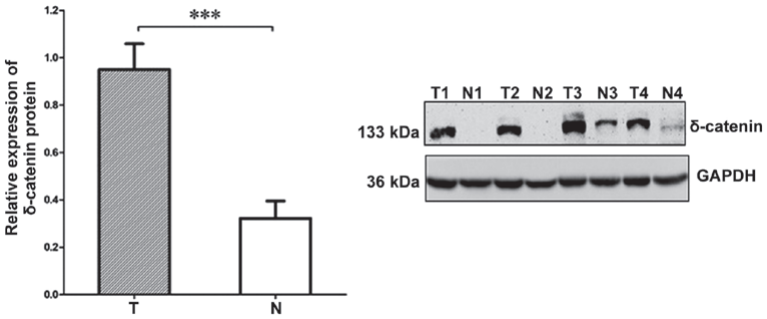
Western blot analysis of δ-catenin protein expression in colorectal cancer tissues. δ-Catenin protein expression was significantly higher in colorectal cancer tissues than that in the matched normal colorectal tissues. Values are expressed as the mean ± standard deviation. ^***^P<0.0001 (n=30). T, tumor tissue; N, normal tissue.

Finally, the δ-catenin protein expression levels were determined in 30 cases of CRC and the paired adjacent normal colorectal tissues using western blot analysis (Fig. 5). The average δ-catenin protein expression in the normal colorectal tissues (0.32±0.07) was significantly lower than that in the matched CRC tissues (0.95±0.11; P<0.05).

## Discussion

Initially, δ-catenin mRNA was considered to be mainly expressed in the brain. δ-Catenin can bind presenilin (5,6) and is considered to be a major adherens junction-associated protein (4,15–18). Subsequent studies revealed that δ-catenin mRNA is also weakly expressed in pancreatic tissues (5). Paffenholz *et al* (19) provided the first evidence of mammalian δ-catenin protein expression in the external limiting membrane of the retina. δ-Catenin mRNA was also shown to be expressed at low levels in a number of tumor cell lines, including PC12 and human neuroblastoma cells; however, these cell types have the capacity for neuronal differentiation (20). δ-catenin has been shown to be upregulated in >80% (55/65) of prostate adenocarcinoma samples, and expression of δ-catenin was demonstrated to be positively correlated with the Gleason score in prostate adenocarcinoma (8). In addition, δ-catenin is expressed at high levels in >60% of patients with non-small cell lung cancer (9), and δ-catenin has been shown to promote a malignant phenotype in non-small cell lung cancer cell by affecting the activity of the transcriptional repressor Kaiso (10).

Analysis of human expressed sequence tags demonstrated that δ-catenin mRNA may be expressed in multiple tumor types, including esophageal, ovarian and breast cancer (21); however, the expression and clinical significance of δ-catenin in numerous tumor types has remained elusive. To determine the role of δ-catenin in CRC, the present study determined the expression of δ-catenin in 110 cases of CRC and 15 adjacent normal colorectal tissues using immunohistochemistry. δ-Catenin was observed to be absent or weakly expressed in the cytoplasm of normal colorectal epithelial cells, which were classified as δ-catenin-negative according to the scoring criteria. Overexpression of δ-catenin was observed in ~70% of the CRC tissues, similar to the results of studies on non-small cell lung cancer (9,10). In addition, semi-quantitative western blot analysis confirmed that δ-catenin protein was overex-pressed in CRC tissues.

The immunohistochemical analysis in the present study demonstrated that δ-catenin was mainly expressed in the cytoplasm of CRC cells, with only small amounts of δ-catenin observed at the cell junctions, in agreement with the localization of δ-catenin in prostate cancer and lung cancer as reported by Lu *et al* (8) and Zhang *et al* (9). It is known that δ-catenin can bind to the juxtamembrane domain of E-cadherin to exert a function in the formation and stability of adherens junctions (15); however, the purpose of the abundance of δ-catenin in the cytoplasm of tumor cells is currently elusive. It is possible that overexpression of δ-catenin may lead to supersaturation of E-cadherin and abnormal accumulation of δ-catenin in the cytoplasm. Alternatively, E-cadherin may lose its function as an adhesion molecule in CRC, and as a consequence, be rarely expressed at the cell membrane. Further study regarding the metabolism of δ-catenin and its association with E-cadherin is required to explain the cytoplasmic expression pattern of δ-catenin in CRC.

Zhang *et al* (9) and Dai *et al* (10) demonstrated that δ-catenin is not an independent prognostic factor in non-small cell lung cancer; however, an association between δ-catenin and poor prognosis was observed. In the present study, over-expression of δ-catenin in CRC was associated with poor differentiation, high TNM stage and lymph node metastasis. The rate of positive δ-catenin expression in lymph node metastases (92.5%; 37/40) was significantly higher than that in the matched primary CRC tumor foci (75%; 30/40). Survival analysis revealed that the mean survival time of CRC patients with positive δ-catenin expression was markedly shorter than that of patients with negative δ-catenin expression, and multivariate analysis confirmed that δ-catenin was an independent risk factor which affected the survival of CRC patients (P=0.031). These results demonstrated that positive expression of δ-catenin is associated with a poorer prognosis in CRC, and indicate that δ-catenin may have an important role in the occurrence and development of colorectal cancer. δ-Catenin should be considered as a potential prognostic factor for predicting the clinical outcome of patients with colorectal cancer.

It has been reported that increased expression of δ-catenin in tumor tissues may be linked to overexpression of the transcription factors E2F1 and Pax6 (22). However, it has also been suggested that the transcription of δ-catenin is not altered in tumor tissues, and that the increased protein expression levels are due to increased translational efficiency (23). In the present study, RT-qPCR analysis demonstrated that the expression of δ-catenin mRNA was significantly increased in CRC tissues compared to that in the matched normal tissues. In addition, the δ-catenin mRNA expression levels were positively correlated with the pathological stage and lymph node metastasis. These results indicated that overexpression of δ-catenin protein in CRC is due to increased transcription of δ-catenin mRNA; however, further study is required to identify the specific mechanisms responsible for this process.

In conclusion, the presents study showed that δ-catenin protein is overexpressed and mainly localizes to the cytoplasm in CRC. Positive expression of δ-catenin was associated with poor differentiation, higher TNM stage, lymph node metastasis and poor prognosis in CRC. Expression of δ-catenin mRNA was upregulated in CRC compared to the corresponding adjacent normal tissues, and the δ-catenin mRNA expression levels were positively correlated with the tumor stage and lymph node metastasis in CRC. Hence, δ-catenin may represent a potentially clinically useful independent prognostic factor in CRC.

## Figures and Tables

**Table I:** Primers and amplification range used in reverse transcription quantitative polymerase chain reaction.

Gene name	Primer sequence sequence (5′→3′)	Amplification range (bp)
δ-catenin	Forward: TTCATCACAGGTGCTGCGTAA	2266–2358
	Reverse: CCATCACACTCTCTCATCCTTCTG	(NM_001332.2)
GAPDH	Forward: GGTGAAGGTCGGAGTCAACG	111–232
	Reverse: CCATGTAGTTGAGGTCAATGAAG	(NM_002046.3)

**Table II:** Clinical and histological features of 110 patients with colorectal cancer.

Variable	All patients (n)	δ-catenin		P-value
		Positive (n)	Negative (n)	
Total	110	75	35	
Age (years)				0.738
<61	54	36	18	
≥61	56	39	17	
Gender				0.775
Male	67	45	22	
Female	43	30	13	
Lesion location				0.686
Rectum	69	48	21	
Colon	41	27	14	
Size (cm)				0.122
<5	67	42	25	
≥5	43	33	10	
TNM stage				0.012
I–II	53	30	23	
III–IV	57	45	12	
Grade				0.012^a^
Well	36	21	15	
Moderate	55	36	19	
Poor	19	18	1	
Lymph node metastasis				0.016
Yes	53	42	11	
No	57	33	24	

a P-value was obtained from the Fisher probabilities, while all others were obtained using the χ^2^ test (two-sided). TNM, tumor, nodes and metastasis.

**Table III:** Cox regression model for the prediction of survival of 70 patients with colorectal cancer.

Factor	β	SE	P-value	Exp (β)	95% CI for Exp (β)
Age	0.012	0.018	0.528	1.012	0.976–1.049
Gender	−0.809	0.461	0.080	0.445	0.180–1.100
Lesion location	−0.511	0.441	0.247	0.600	0.253–1.425
Tumor size	−0.408	0.473	0.388	0.665	0.263–1.680
Grade	0.149	0.307	0.626	1.161	0.636–2.119
TNM stage	1.598	0.608	0.009	4.945	1.502–16.281
Lymph node metastasis	−0.948	0.669	0.156	0.387	0.104–1.437
δ-catenin expression	1.113	0.515	0.031	3.042	1.109–8.343

SE, standard error; CI, confidence interval; Exp (β), odds ratio; TNM, tumor, nodes, metastasis.
